# Case report: A rare case of malignant solitary fibrous tumor in an adult with an epithelioid pattern in the occipital region

**DOI:** 10.3389/fonc.2024.1339582

**Published:** 2024-08-16

**Authors:** Ke Huang, Wen-wen Liu, Xiu-wen Chen, Yin-hua Hao, Sen-yuan Luo, Ling-ling Yuan, Yu-gang Huang, Xian-bin Tang

**Affiliations:** Department of Pathology, Taihe Hospital, Hubei University of Medicine, Shiyan, China

**Keywords:** case report, malignant solitary fibrous tumor, NAB2-STAT6 fusion, diagnostic pathology, Next-generation sequencing-NGS

## Abstract

We illustrated a rare case of malignant solitary fibrous tumor (MSFT) with epithelioid morphology in the occipital region of a 59-year-old female, in which a rare NAB2ex7-STAT6 exon15/16 double fusion subtype was detected by the Next-generation sequencing (NGS) and STAT6 immunohistochemistry (IHC) was diffusely and strongly positively expressed, without recurrence after 20 months of postoperative follow-up. The morphological and molecular genetic aspects and the differential diagnosis are described, and the relevant literature was assessed in order to broaden our understanding and diagnostic capability of this malignancy.

## 1 Introduction

Malignant solitary fibrous tumor (MSFT) is a rare malignant mesenchymal carcinoma that may occur throughout the body (1). Unlike the typical spindle cell morphology of SFT (2), MSFT cells have a degree of heterogeneity, may occasionally exhibit epithelioid or sarcomatoid differentiation and are purely composed of epithelioid cells (3, 4).

The diagnosis is more difficult since the morphology of this case is particularly rare, microscopically composed entirely of epithelioid cells, lacking the characteristic fibroblastic spindle cell population of SFT, and occurring in a more specific location, located in the occipital subcutaneous area rather than the chest or meninges, in which SFT is well known. SFT was not considered at the point of the initial diagnosis. Because of immunohistochemical CD99 diffuse membrane positivity, we performed a FISH test for EWSR1 to identify extraosseous Ewing sarcoma (5), and the count was positive, but FLI-1 immunohistochemistry negativity put the diagnosis in an awkward position. Thus, we utilize the NGS and RT-PCR to explore the molecular characteristics of the tumor and probe accurate evidence for pathological diagnosis. Written informed consent for this study was obtained from the patient herself.

## 2 Case presentation

**Figure 1:**
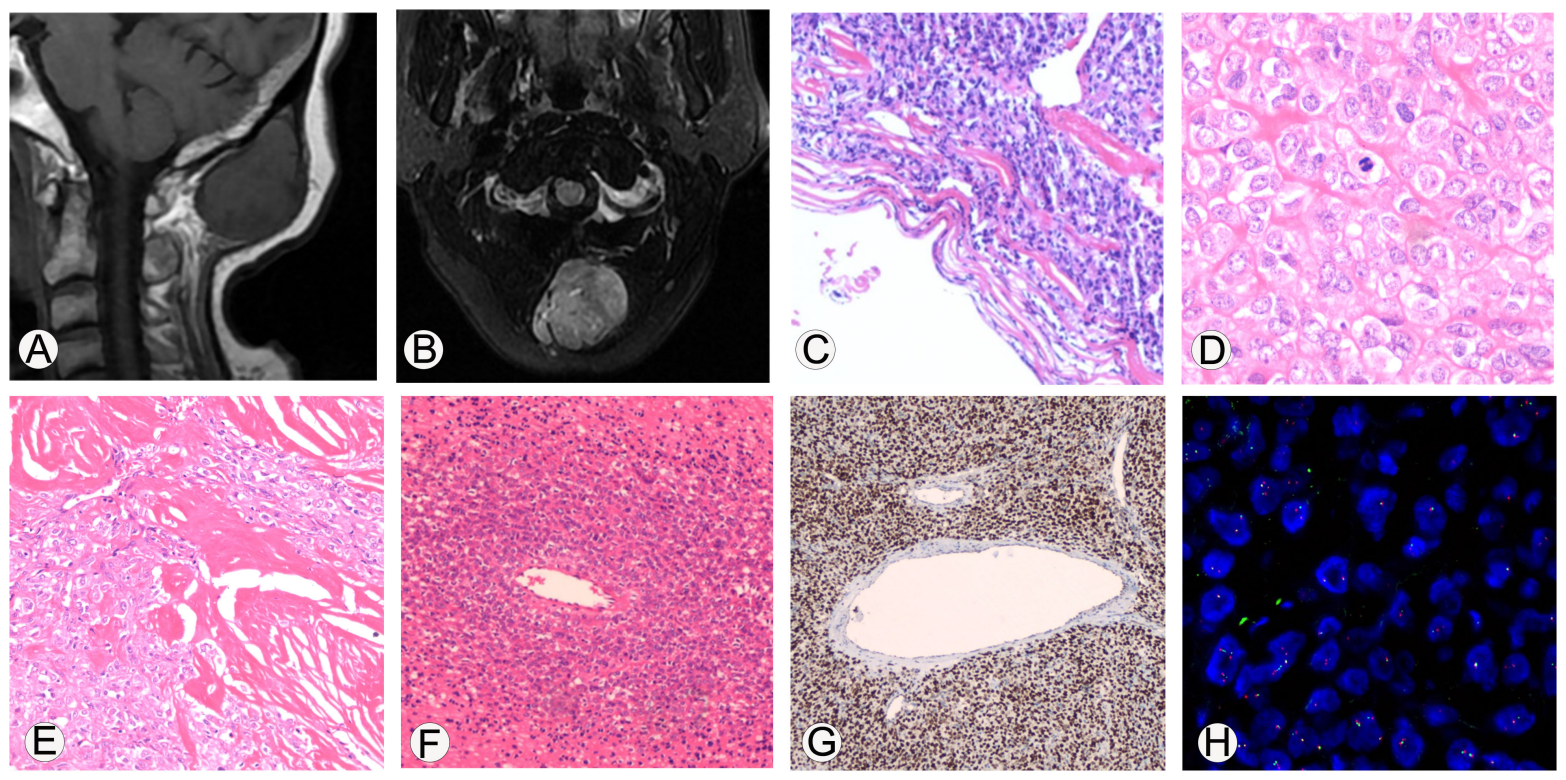
Imaging and immunohistochemical staining related to the pathological diagnosis of malignant solitary fibrous tumor. **(A, B)**, MRI shows that the tumor is located subcutaneously in the occipital region, with clear boundaries. On T1WI, it shows iso-low signal intensity, while on T2Flair, it shows mixed high signal intensity. **(C)**, Microscopic observation shows that the boundary of the tumor was clear and appeared to have a fibrous pseudo capsule; 100x magnification. **(D)**, The tumor cells are epithelioid with eosinophilic cytoplasm, and the stroma in some areas was hyaline; 400x magnification. **(E)**, Collagen deposition can be seen between tumor cells, resembling sclerosis, and some areas of collagen fibers show nodular growth; 200x magnification. **(F)**, necrosis can be seen in the tumor, and dense tumor cells can grow around blood vessels around the necrotic area; 100x magnification. **(G)**, Immunohistochemical analysis showed diffuse strong expression of STAT6 protein in tumor cells; 100x magnification. **(H)**, EWSR1 FISH detection showed that the proportion of cells with red-green separation signals was about 22%.

A 59-year-old female presented to Taihe Hospital more than a year after the discovery of an occipital mass. Physical examinations showed a mass in the occipital region, approximately 3.0*3.0 cm, with soft texture, no pressure pain, and general mobility. Magnetic resonance imaging (MRI) of the head showed that a well-defined nodule was visible in the occipital subcutaneous region, with an iso-low signal in T1WI (**Figure 1A**) and mixed high signal in T2Flair (**Figure 1B**). There was no previous history of cancer. Under general anesthesia, the patient had an occipital soft tissue lesion resected. The tumor was discovered to have clear borders with intact peripheral membranes attached to the surface during surgery, and it grew downward, tightly attached to the skull, and penetrated deeper into the occipital bone near the greater occipital foramen, which was appropriately resected after complete stripping of the tumor.

The histopathological investigation of the removed mass revealed the following. (1) Gross examination: one nodule, 4.0 cm×3.8 cm×3.3 cm, encased in a capsule, with a gray-white solid texture on the cut surface. (2) Microscopic examination: the boundary of the tumor was clear and appeared to have a fibrous pseudo capsule (**Figure 1C**); the tumor cells were epithelioid, the cytoplasm was eosinophilic, and the stroma in some areas was hyaline (**Figure 1D**); the nuclei of the tumor cells were moderately heterogeneous, the nuclei were vacuolated, small nucleoli were visible, and the nuclear pleomorphism and mitosis were about 6/10 high power field (HFP). Collagen deposition was visible between tumor cells, as well as sclerosis, with collagen fibers forming in a knot-like pattern in one region (**Figure 1E**). Tumor cells in other regions became degenerated and necrotic, and tumor cells in the vicinity of the necrotic areas were shown to develop densely around the blood vessels (**Figure 1F**). Immunohistochemical analysis showed diffuse strong expression of STAT6 protein in tumor cells (**Figure 1G**), CD99, Vimentin, INI1, Calponin, BCOR, and focally positively expressed CK, CD34, SMA, and sporadically expressed TLE1, and did not express CK20, P63, FLI-1, NKX2.2, S100, Myoegnin, WT-1, CyclinD1, SATB2, CD3, CD20, CD43, and Ki-67 value-added index of about 10%.

**Figure 2:**
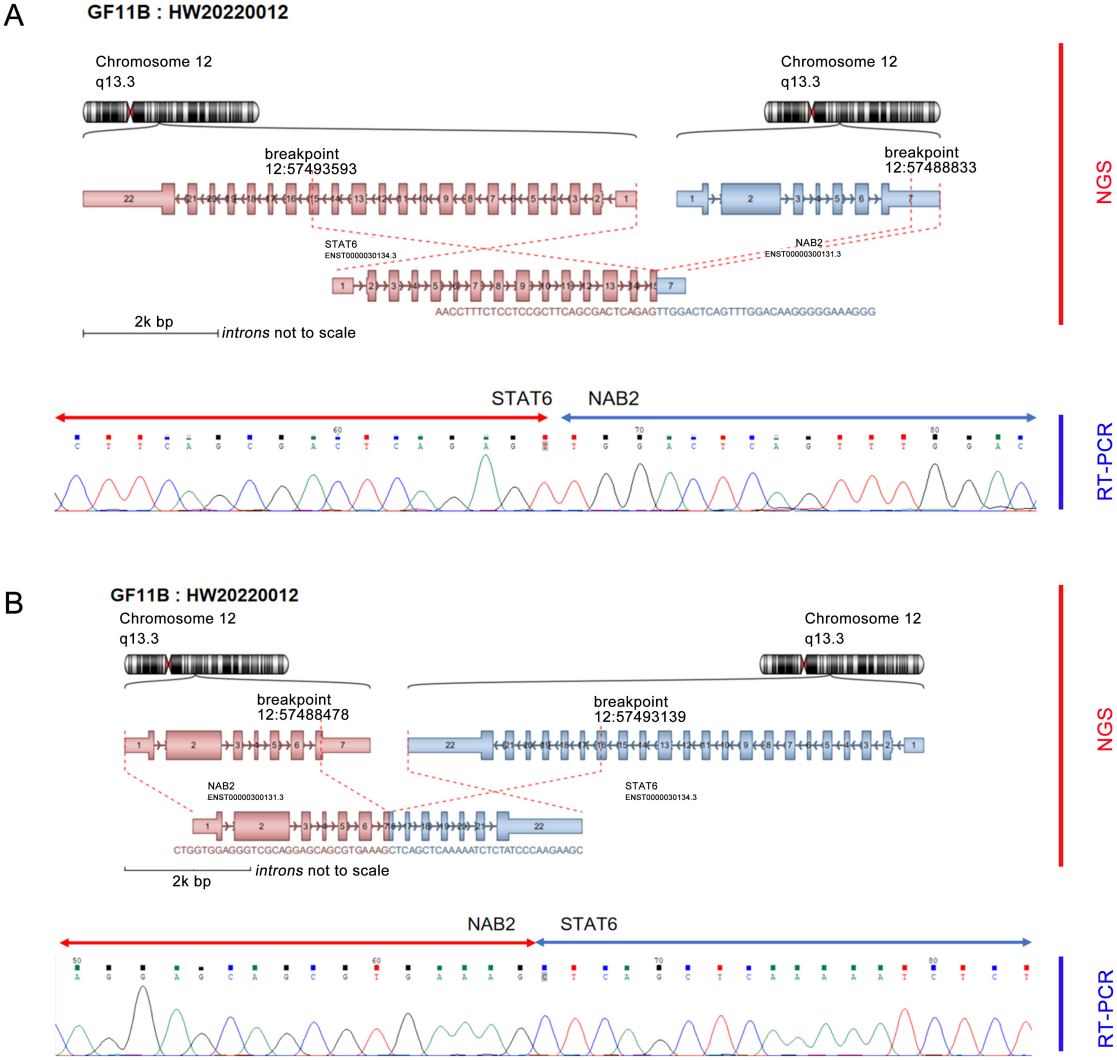
NGS detected dual fusion of the NAB2-STAT6 gene, including STAT6 exon 15-NAB2 exon 7 **(A)** and NAB2 exon 7-STAT6 exon 16 **(B)**.

Furthermore, the molecular pathology examination suggested that the EWSR1 FISH assay count was positive (the percentage of cells with red and green separated signals was about 22%, **Figure 1H**). RNA-seq detected the fusion of the NAB2 and STAT6 genes and the presence of two fusion isoforms at the same time, namely, STAT6ex15-NAB2ex7 (**Figure 2A**) and NAB2ex7-STAT6 exon 16 (**Figure 2B**). (NAB2: NM_005967.3, STAT6: NM_003153.4).

Our final pathologic diagnosis was a malignant isolated fibrous tumor with an epithelioid pattern, medium risk.

At 20 months of follow-up, the patient had undergone surgical subcutaneous full excision of the tumor, with good postoperative recovery, no radiation, and no tumor recurrence or metastasis.

## 3 Discussion

The morphology of this case was very rare, occurring in a rather unusual location, subcutaneous in the occipital region (rather than in the thorax or meninges, where SFT is favored), and microscopically consisted exclusively of epithelioid cells, lacked the fibroblastic spindle cell population characteristic of SFT, and in addition was diffusely positive for CD99 (6). The only morphological and immunohistochemical findings confounded the pathologic diagnosis. Therefore, the NGS assay was performed to screen two kinds of NAB2-STAT6 fusion genes. RT-PCR successfully identified the result and also detected the fusion of exon 10 of the EWSR1 gene and exon 5 of the PBX1 gene. Still, the number of detected Split Reads was too low to be effectively detected by the RT-PCR method due to its sensitivity, and this fusion form needs to be further verified. Previous studies reported that NAB2-STAT6 fusion was a characteristic molecular genetic alteration biomarker of SFT (3, 7), which has not been detected in other diseases, and based on it, we added relevant immunohistochemistry. We found that STAT6 was diffusely and strongly positively expressed in tumor cells, which IHC also supported.

The management and prognosis of SFT is different from that of other malignant tumors, making proper diagnosis crucial. Although SFT expresses a unique immunohistochemical (IHC) profile, classical histomorphologic and IHC profiles are not seen in all cases, and diagnosis can be challenging. The NAB2-STAT6 gene fusion has recently emerged as a sensitive and specific molecular marker for SFT, and its specific transcriptional activator 6 (STAT6) has also shown significant sensitivity and specificity (8). STAT6 nuclear positivity in tumor cells is important for the diagnosis of SFT, with a sensitivity of 95. 8% to 100% and a specificity of 88. 3% to 100% (9).

STAT6 was found to be diffusely and strongly positively expressed in tumor cells, which also supported the diagnosis of SFT (10, 11). In the literature, Suster et al. (12) reported 13 cases of epithelioid SFT, which were composed entirely of epithelioid cells under the microscope, and Argani et al. (13) declared 5 cases of MSFT occurring in the kidneys, which were also characterized by predominantly epithelioid cells, with some of the epithelioid cells in a hyaline state. In this case, the morphology was also composed of epithelioid cells, some of which were hyaline, and was consistent with the basis of malignancy, leading to the diagnosis of epithelioid MSFT. The NGS assay contributed decisively to the diagnosis of this case, re-emphasizing the importance of molecular testing.

SFTs have a diverse morphology and can present as round cells, giant cells, mucus-like, pleomorphic, and dedifferentiated, in addition to the classic spindle cell morphology (14, 15). The fourth edition of the WHO Classification of soft tissue and bone tumors introduced the concept of malignant isolated fibrous tumors: it also meets the criteria of increased tumor cell density, active nuclear division (>4/10HFP), cells with heterogeneity, tumor necrosis, and or infiltration of the margins (16). In this case, the increased density of tumor cells on microscopy, nuclear division like about 6/10HFP in the proliferative active area, cells with moderate heterogeneity, and tumor degeneration and necrosis in some areas meet the diagnostic criteria of MSFT (17, 18).

NAB2-STAT6 is a characteristic fusion of SFT and can be detected in 55% to 100% of SFT and 100% of MSFT (19). There are three most common fusion subtypes: NAB2 exon 6-STAT6 exon 16/17 versus NAB2 exon 4-STAT6 exon 2, and different fusion subtypes have different clinicopathologic features (8). SFT patients who develop NAB2ex4-STAT6ex2 fusion are usually older, and the tumors are mostly located in the chest, large in size, less likely to recur, and microscopically seen to be sparsely populated with low mitotic activity and abundant fibrosis (14). In contrast, SFT patients who develop NAB2 exon 6-STAT6 exon16/17 fusion are usually younger, the tumors are often located in the deep inner soft tissues, such as the abdomen, retroperitoneum, pelvis and other regions, with a higher recurrence rate, and microscopically, the cells are usually rounded to ovoid in shape with higher mitotic activity (20). As for the fusion subtype that occurred in this case, the fusion subtype was NAB2 exon 7-STAT6 exon 16 in only one of the 44 cases of SFT in the meninges reported by Bieg et al. (21). The occurrence of two rare fusion subtypes at the same time, such as in this case, has not been similarly reported so far.

Currently, over 40 different mutation types have been identified in SFTs (22), and only one of the double fusion isoforms that occurred in this case is identical to NAB2ex7-STAT6ex16 in one of the 44 cases of SFTs reported by Bieg et al. (21). The occurrence of two rare fusion isoforms at the same time has not been similarly reported.

The term “epithelioid SFTs” was first proposed by Alberto et al. in 2003 (23), who reported a mediastinal SFTs with predominantly epithelioid cells, accompanied by a spindle cell component; since then, SFTs accompanied by epithelioid cells have been consistently reported, but SFTs composed exclusively of epithelioid cells are extremely rare. In the reported literature, Mourra et al. (24) have reported a case of benign SFTs occurring in the sciatic fossa composed entirely of epithelioid cells; Yan et al. (25) reported a case of malignant epithelioid SFTs of the pleura, which was a primitive case with marked cellular anisotropy; Jing et al. (26) reported a recurrent SFTs occurring in the meninges, where the tumor showed a typical benign at the time of primitive SFT morphology, and the recurrent tumor consisted entirely of epithelioid cells; Suster et al. (12) had reported 13 cases of SFTs with epithelioid morphology, of which the tumors were located in 3 cases in the orbit, 3 cases in the lower limbs, 2 cases in the retroperitoneum, 2 cases in the abdominal cavity, and 1 case each in the superficial soft tissues of the neck, pelvic affixation of the bladder, and the pubic bone, with most cases having a mild morphology of the epithelioid cells, and a few showing a mild increase in cytological atypia; Argani (13) et al. reported five cases of malignant SFTs occurring in the renal and pararenal regions, with morphologic and immunologic features that clearly overlap with renal clear cell sarcoma; the tumors consisted of round or ovoid primitive undifferentiated morphologic cells, lacked areas of benign SFTs, and the BCOR showed diffuse nuclear reactivity. In our case, the tumor was located subcutaneously in the occipital region and consisted exclusively of epithelioid cells with moderately heterogeneous nuclei, increased nuclear fission, and visible necrosis, which was histologically consistent with malignant SFT; furthermore, immunohistochemistry BCOR in this case showed a diffuse and strong positivity, which is in line with previous reports of malignant epithelioid SFTs; related studies have shown that BCOR immunohistochemistry positivity is associated with fusion variants in SFTs, tumor size and location; a recent report described that BCOR was upregulated in NAB2ex6-STAT6ex16/17 cases than in NAB2ex4-STAT6ex2 cases (21), and its positivity was more commonly seen in malignant SFTs (13), suggesting that BCOR might be useful as a judgmental indicator of malignant SFTs.

SFTs with complete epithelioid morphology can be easily misdiagnosed and needs to be distinguished from the following diseases: (1) Epithelioid sarcoma (ES): a malignant tumor with the unclear direction of tumor cell differentiation, clinically classified into classic and proximal types, occurring in the limbs of young patients, and is a superficial soft-tissue tumor (27); morphology reveals epithelioid cells, with immunohistochemistry of cytokeratins positive expression, molecular detection, and a lack of SMARCB1 (28). The absence of SMARCB1 can be differentiated from epithelioid SFTs. (2) Sclerosing epithelioid fibrosarcoma (SEF): SEF is a rare soft tissue tumor in which a large number of collagenous mesenchyme with vitreous metaplasia and polygonal epithelioid tumors interspersed with striated cells are seen microscopically, which overlap morphologically with epithelioid SFTs (29). Compared with SFT, SEF is positive for MUC4 and negative for STAT6 (30), and the FUS gene is often rearranged (31). (3) Meningioma or malignant meningioma: When epithelioid SFTs are located in the orbital region, they need to be differentiated from meningiomas/malignant meningiomas, which can be distinguished using immunohistochemistry such as STAT6 (32). (4) Clear cell sarcoma (CCSK): some malignant epithelioid SFTs occurring in the kidney can be morphologically similar to CCSK (33), especially when the BCOR immunohistochemistry of the SFTs shows diffuse strong positivity, which can be easily misdiagnosed, and needs to be distinguished by relying on molecular tests *etc.* (34, 35). (5) Other tumors that may present epithelioid cell morphology, such as synovial sarcoma (36), angiosarcoma (37), smooth muscle sarcoma (38, 39), malignant peripheral nerve sheath tumor (40), gastrointestinal mesenchymal stromal tumor (41), and mucoinflammatory fibroblastic sarcoma (42). (6) Dedifferentiated and trans-differentiated SFT: Dedifferentiated SFT refers to tumors with areas of poorly differentiated epithelioid cells, round cells, or spindle-shaped high-grade sarcoma cells in addition to benign SFT areas, both of which usually show abrupt migration, and the dedifferentiated areas are usually accompanied by loss of expression of CD34, attenuated or absent expression of STAT6, and strong expression of p53 and p16 (4, 43). Transdifferentiated SFT is a tumor that presents with an abnormal phenotype distinct from its SFT origin, possibly associated with high-grade transformation or dedifferentiation of the tumor, with the transdifferentiated component usually accounting for most if not all of the tumor, and the classical SFT component is often difficult to find (44). Both dedifferentiated and transdifferentiated SFT can result in the presence of CK-positive epithelial components in the tumor through mesenchymal-epithelial transformation (14). Therefore, when epithelioid morphology is present in SFT, dedifferentiated or transdifferentiated SFT needs to be excluded.

SFT has a certain risk of recurrence and metastasis. Salas S et al. found that 20 patients (12.3%) had local recurrence and 27 patients (16.7%) had metastatic recurrence through a study and analysis of 162 patients with SFT. It revealed that whether postoperative radiotherapy was supplemented or not was a key factor affecting the prognosis of SFT, and radiotherapy was effective in reducing the recurrence and metastatic rates of SFT (45). Since pathological features may not always fully reflect clinical aggressiveness, a stratification model for predicting the risk of metastasis of SFTs was proposed in the fifth edition of the WHO Classification of Soft Tissue and Bone Tumors, which classified the risk of metastasis as low, intermediate, or high based on the tumor size, the extent of necrosis, the mitotic count, and the patient’s age (46). Currently, the predominant treatment approach is individualized treatment for people with different levels of metastatic risk. For people with high risk of metastasis, radiotherapy can reduce the recurrence rate of metastasis (8, 45).

The metastatic risk in our case was intermediate, and we have been followed up for 20 months after complete resection and are still surviving disease-free. Currently, the treatment of MSFT is based on complete resection of the tumor, supplemented by radiotherapy when necessary, according to the situation (47, 48). Recently, it has been found that when MSFT has telomerase reverse transcriptase gene (TERT) promoter mutations, TP53 gene mutations, and APAF1 gene mutations, the tumor is more aggressive, with a relatively high risk of metastasis and a relatively poorer prognosis (49). For such aggressive MSFT, in addition to conventional surgical resection, subsequent chemotherapy combined with targeted drugs such as Bevacizumab, Pazopanib, Sunitinib, and Tazemetostat can be used (50–52). However, for patients who have already experienced recurrence or metastasis, there has not yet been a definitive approach to the treatment of SFT, which still needs to be further explored.

As for epithelioid SFTs, Jing Fu et al. (26) found that a case of SFTs with a few epithelioid cells in the admixture of epithelioid cells developed malignant transformation of epithelioid cells 68 months later, and therefore suspected that epithelioid features might be associated with more aggressive clinical behaviors, whereas David I et al. concluded that tumors with this appearance should be classified as normal by analyzing 13 SFTs with epithelioid morphology. SFTs should be categorized as low, intermediate and high risk based on risk stratification as well (12). Since cases of epithelioid SFTs are very rare, more cases need to be studied at this time to draw definitive conclusions about the prognostic significance of these features.

## 4 Conclusions

In conclusion, we report a case of MSFT with epithelioid morphology occurring in the occipital region of a 59-year-old female. The tumor was located in an unusual location, subcutaneously in the occipital region, not in the chest or meninges, where SFT occurs; the tumor cells were not of the classic spindle cell morphology but consisted of purely epithelioid cells, which is very rare. The NGS detected a rare double fusion isoform of NAB2ex7-STAT6ex15/16, and the STAT6 immunohistochemistry showed a diffuse positive expression. STAT6 was diffusely and strongly positively expressed by immunohistochemistry. This completely epithelioid morphology of SFT is very easy to misdiagnose, and attention needs to be paid to differential diagnosis with other diseases (including ES, SEF, meningioma or malignant meningioma, CCSK, *etc.*). Precise treatment of neoplasms requires precise diagnosis of pathology, and the combination of multiple technological assays provides an effective avenue for the implementation of precise diagnosis.

## Data Availability

The original contributions presented in the study are included in the article/supplementary material. Further inquiries can be directed to the corresponding author/s.
